# Highlighting Fibroblasts Activation in Fibrosis: The State-of-The-Art Fibroblast Activation Protein Inhibitor PET Imaging in Cardiovascular Diseases

**DOI:** 10.3390/jcm12186033

**Published:** 2023-09-18

**Authors:** Yan Cui, Yuxiang Wang, Shu Wang, Bulin Du, Xuena Li, Yaming Li

**Affiliations:** Department of Nuclear Medicine, The First Hospital of China Medical University, Shenyang 110001, China; cy3893096@163.com (Y.C.); chriswyx0120@163.com (Y.W.); shuwang201508@163.com (S.W.); dubulin@163.com (B.D.)

**Keywords:** fibroblast activation protein (FAP), FAP inhibitor (FAPI), positron emission tomography (PET), fibroblast, fibrosis, cardiovascular disease

## Abstract

Fibrosis is a common healing process that occurs during stress and injury in cardiovascular diseases. The evolution of fibrosis is associated with cardiovascular disease states and causes adverse effects. Fibroblast activation is responsible for the formation and progression of fibrosis. The incipient detection of activated fibroblasts is important for patient management and prognosis. Fibroblast activation protein (FAP), a membrane-bound serine protease, is almost specifically expressed in activated fibroblasts. The development of targeted FAP-inhibitor (FAPI) positron emission tomography (PET) imaging enabled the visualisation of FAP, that is, incipient fibrosis. Recently, research on FAPI PET imaging in cardiovascular diseases increased and is highly sought. Hence, we comprehensively reviewed the application of FAPI PET imaging in cardiovascular diseases based on the state-of-the-art published research. These studies provided some insights into the value of FAPI PET imaging in the early detection of cardiovascular fibrosis, risk stratification, response evaluation, and prediction of the evolution of left ventricular function. Future studies should be conducted with larger populations and multicentre patterns, especially for response evaluation and outcome prediction.

## 1. Introduction

Fibrosis is a common response and an important mediator of various types of injuries involved in cardiovascular diseases. Although fibrosis may be sometimes beneficial and crucial for preventing dysfunction of the involved organs in some circumstances (myocardial infarction, vascular diseases, etc.), as the disease evolves, fibrosis gradually becomes uncontrolled and excessive, contributing to adverse cardiovascular remodelling and development of adverse cardiovascular events [1,2]. Therefore, unsuitable and exaggerated fibrosis should be focused and evaluated [3]. However, identification of this detrimental fibrosis remains challenging and deserves more investigation on early detection, precise stratification, and prediction of the evolution of left ventricular (LV) function of cardiovascular diseases [4].

There are multiple imaging modalities for evaluating cardiovascular fibrosis, such as echocardiography, cardiac magnetic resonance (CMR), and [^18^F]F-fluorodeoxyglucose (FDG) positron emission tomography (PET)/CT, none of which are specific to fibrosis [5]. Echocardiography is the most common cardiac imaging technique because it is economic and accessible, focusing on structural and functional abnormalities in cardiovascular diseases instead of specific fibrosis [6]. CMR imaging reflects extracellular infiltration and deposition rather than fibrosis itself [7]. [^18^F]F-FDG PET/CT focuses on viable myocardium rather than fibrosis [8]. These imaging modalities reflect fibrosis-related issues such as changes in extracellular matrix (ECM) components, rather than focusing on the central cellular effectors of fibrosis and fibroblasts. Therefore, a specific biomarker for detecting cardiovascular fibrosis is required. 

Activated fibroblasts (myofibroblasts expressing alpha smooth muscle actin [α-SMA]) are involved in extracellular remodelling in cardiovascular diseases by influencing ECM degradation and collagen synthesis [9]. These characteristics render activated fibroblasts crucial for both the pathogenesis and repair of adverse cardiac remodelling. Fibroblast activation protein (FAP), a membrane-bound serine protease, is highly expressed in activated fibroblasts; therefore, targeted FAP inhibitor (FAPI) radiopharmaceuticals were developed to visualise activated fibroblasts [10]. These FAPI tracers are widely used in PET/CT imaging in the oncological field for the imaging of targeting cancer-associated fibroblasts [11]. Meanwhile, a retrospective analysis of [^68^Ga]Ga-FAPI-04 imaging in oncologic cohorts found an association between FAPI uptake and cardiac risk factors [12]. Increased cardiac [^68^Ga]Ga-FAPI uptake was also reported after chemoradiotherapy in oncologic patients [13]. Thus, this novel imaging approach may provide a more specific visualisation and evaluation of activated fibroblasts and fibrosis in cardiovascular diseases. 

In brief summary, fibrotic response to various cardiovascular etiologies is regulated by activated fibroblasts. FAP is a specific biomarker for activated fibroblasts, rendering FAPI PET imaging the capability of visualising fibrosis in cardiovascular diseases. Moreover, unsuitable and exaggerated fibrosis should be considered as the theranostic target. This review summarises and discusses the state-of-the-art applications and the performance of FAPI PET imaging in various cardiovascular diseases by highlighting fibroblast activation in fibrosis for early detection, risk stratification, response evaluation, and prediction of the evolution of LV function of cardiovascular diseases, providing some clues for further investigation and facilitating the wide utilisation of this approach.

## 2. Fibrosis in Cardiovascular Diseases

Fibroblasts, a vital cellular element in fibrosis, are defined as cells of mesenchymal origin without a basement membrane that produce ECM proteins to form and maintain connective tissues [14]. Healthy fibroblasts are widespread and the majority of them are physiologically quiescent. These resting fibroblasts can be activated in a context-dependent manner such as during wound healing and inflammation in cardiovascular diseases [15]. Once activated, fibroblasts in the heart and vessel differentiate into profibrotic phenotypes, defined as myofibroblasts, secreting more extracellular matrices, and participating in the subsequent formation of a collagen scar [16,17]. As the scar matures, ECM remodelling and fibrosis occur, followed by cardiac dysfunction and vascular abnormality [18].

### 2.1. Fibrosis in Cardiac Diseases

Under normal conditions, cardiomyocytes hold nearly 75% of the myocardial volume in humans and are surrounded by the interstitial ECM. The ECM is composed of type I collagen, type III collagen, glycoproteins, proteoglycans, growth factors, and proteases that can be activated by injury and stimulation. Excessive deposition of ECM proteins in cardiac parenchymal tissues under various pathological conditions is defined as myocardial fibrosis. Myocardial fibrosis can be classified into two types: reactive fibrosis and replacement fibrosis, both of which are mediated by fibroblasts and myofibroblasts [19].

In the early stages of cardiovascular diseases, reactive fibrosis occurs and is characterised by the deposition of ECM proteins without significant cardiomyocyte death. Mechanistically, reactive fibrosis is often associated with diffuse myocardial injury, including pressure overload. Impaired ventricular compliance and diastolic dysfunction were observed in patients with reactive fibrosis. After the removal of these stimuli, reactive fibrosis may be reversed, whereas it may be exacerbated into replacement fibrosis if the stress persists [20].

Replacement fibrosis is defined as the replacement of necrotic cardiomyocytes with collagen scars. Although its molecular mechanism and structural composition are similar to reactive fibrosis, unlike in reactive fibrosis, replacement fibrosis, which often presents with focal involvement, is irreversible, and associated with impaired systolic dysfunction [21,22]. There are no obvious boundaries between the two types of fibrosis, which even overlap and coexist during complicated and long-lasting fibrogenesis [23].

Cardiac fibrosis is a complex process that involves multiple cell types, cytokines, and proteins. Immune cells such as macrophages, lymphocytes, and mast cells may activate fibroblasts by secreting cytokines and structural ECM proteins. Vascular endothelial cells and pericytes promote fibroblast activation and serve as myofibroblast precursors [3]. Myofibroblasts contribute to cardiac fibrosis as follows: Firstly, various pro-inflammatory and pro-fibrotic elements are increasingly secreted by myofibroblasts, resulting in inflammatory cell recruitment and fibroblast proliferation. Secondly, diverse ECM-degrading enzymes are generated by myofibroblasts, which promote their migration. Lastly, structural ECM proteins are produced by myofibroblasts, stimulating collagen synthesis, and eventually fibrosis [20].

### 2.2. Fibrosis in Vascular Diseases

Various triggers, such as inflammation, stress, thrombus formation, and injury, may result in vascular damage, tissue reconstruction, and ultimately, fibrosis. Mechanically, distinct types of cells infiltrate wounded areas and experience pro-fibrotic phenotype transmission, secreting ECM (including collagen type I and fibronectin) to strengthen the involved tissue and promote the repair [24]. Specifically, when a vessel is initially damaged, the cell contents and damage-associated molecules are released, followed by the adjacent activation of mural and mesenchymal cells. Macrophages and monocytes are then recruited to the corresponding areas. Necrotic cells are phagocytosed by macrophages, which also secrete chemokines to attract other leukocytes such as neutrophils. Macrophages also contribute to the transformation of fibroblasts into myofibroblasts [25]. Myofibroblasts play a central role in fibrosis and exhibit contractile and secretory functions. With the persistence of triggers, fibrotic collagen is deposited, fibrosis becomes uncontrolled, damaged tissue is altered, and organ dysfunction occurs [26].

## 3. Targeting FAP

### 3.1. FAP in Cardiovascular Diseases 

The early detection of cardiovascular fibrosis requires a specific biomarker. FAP may play this role [27]. FAP is a type-II integral membrane-bound serine protease. As a member of the dipeptidyl peptidase (DPP) family, FAP embraces both DPP and endopeptidase activities that are involved in various aspects of biology and targeted therapy [28]. Endopeptidase activity is the basis for the FAP-specific detection of inhibitory molecules. The substrates of the FAP endopeptidase activity include denatured collagen types I and III (components of gelatin) [29,30], α2-antiplasmin (α2-AP) cleaving enzyme, and fibroblast growth factor 21 [31]. During tissue repair, fibrin is deposited and forms fibrin clots. Fibrin clots can dissolve in plasmin, resulting in fibrinolysis and scar resolution. α2-AP, an inhibitor of plasmin, can be cleaved and further strengthened by FAP [32]. Therefore, the protein structure and functional characteristics endow FAP with a vital role in fibrosis. Based on current research, FAP is found during embryonic development, granulation tissue formation, and haematopoiesis rather than in healthy tissues. In cardiovascular diseases, FAP is expressed in activated rather than quiescent fibroblasts [33].

Several pre-clinical studies investigated the role of FAP in cardiovascular diseases. FAP expression in the infarction and peri-infarction areas increase, peaking at 7 days after myocardial infarction (MI) in rats, and FAP-expressing fibroblasts were enriched in the ischaemic region of human hearts after MI [34]. Thus, FAP is a marker of fibroblast activation, and FAP expression may indicate a pro-fibrotic phenotype of cardiac fibroblasts in MI. In a recent study targeting cardiac fibrosis with engineered T cells, FAP showed the greatest upregulation of all fibroblast-specific genes when comparing hypertrophic cardiomyopathy (HCM) samples to controls. They demonstrated that FAP is highly expressed by cardiac fibroblasts (not myocytes) in the LV tissue of failing human HCM hearts [35]. In vascular diseases, a recent study found that deprivation of FAP provides atheroprotection in mouse models, and increased FAP expression is observed in human atherosclerotic arteries compared to non-atherosclerotic arteries [36]. These findings indicate that FAP expression is associated with plaque vulnerability and that FAP may serve as a potential target for atherosclerosis imaging non-invasively. 

**Figure 1:**
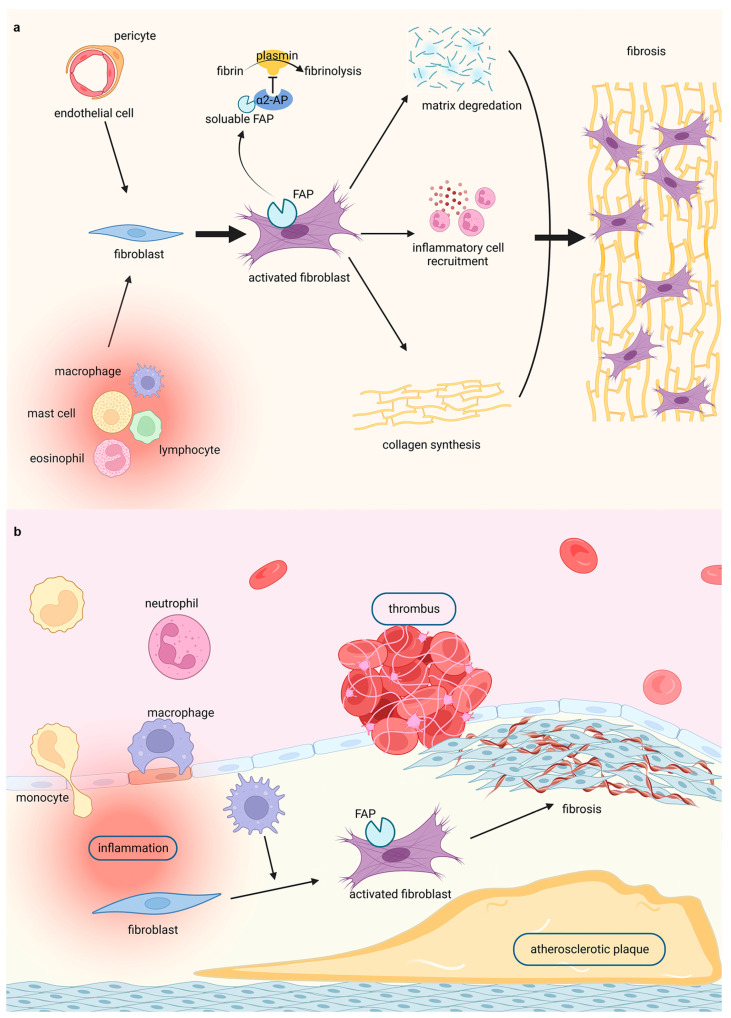
Highlighting fibroblasts activation in fibrosis by FAPI PET imaging in (**a**) cardiac diseases and (**b**) vascular diseases. Figure 1 was created with biorender.com.

In summary, the evidence mentioned above motivates the utilisation of FAP as promising targets for the visualisation and evaluation of fibrosis in cardiovascular diseases. Thus, FAP-targeted molecular imaging could highlight the significance of FAP in cardiac fibrosis (Figure 1a) and vascular fibrosis (Figure 1b).

### 3.2. FAPI Radiopharmaceuticals

#### 3.2.1. Development of FAPI Tracer

FAP targeting radiopharmaceuticals attracted considerable attention. The first clinical investigation of FAP targeting radiopharmaceuticals was a radiolabelled FAP antibody in 1994, followed by a series of studies on radiolabelled FAP targeting antibodies and peptides [37,38,39]. As small-molecule drugs with better tissue penetration and pharmacokinetics emerged, UAMC-1110 emerged as a representative small enzyme inhibitor specific for FAP in 2014 [40]. FAPI was first designed and synthesised based on the 6-quinolyl modification of UAMC-1110, which presents a rapid and almost complete internalisation of the ligand–receptor complex [41]. More FAPI tracers were developed to improve target-to-background ratio (TBR) and image contrast. FAPI-04, published in 2018 by Lindner et al., showed slow excretion and high tumour accumulation, and was considered the best tracer for FAP-targeted radioligand therapy despite its short tumour retention time. Currently, FAPI-04 is the most clinically investigated tracer, with excellent pharmacokinetics and theranostic potential [42]. FAPI-46 was further developed to prolong tumour retention and improve diagnostic ability by replacing the bridging oxygen with methylated nitrogen at the 6-quinoline position [43]. Compared to the classical molecular tracer [^18^F]F-FDG, FAPI presents a better TBR under many conditions and does not require fasting or blood glucose restriction. Moreover, radiolabelled FAPI enables radioligand therapy using a theranostic method. Currently, the application of FAPI tracers in cardiovascular diseases raised significant concerns. [^68^Ga]Ga-FAPI-04, [^68^Ga]Ga-FAPI-46, and [^18^F]AlF-NOTA-FAPI-04 are mainly used FAPI tracers in cardiovascular diseases, including myocardial infarction, coronary artery disease (CAD), heart failure (HF), secondary myocardial injury such as oncotherapy-induced myocardial injury and iatrogenic myocardial injury, cardiac amyloidosis, cardiomyopathy, and vascular diseases such as atherosclerosis, thrombosis, and arteritis. These studies provide the value of FAPI PET imaging in the preliminary evidence, early detection of cardiovascular fibrosis, risk stratification, response evaluation, and prediction of the evolution of LV function through pre-clinical studies, case reports, retrospective studies, and prospective studies, as presented in Table 1.

#### 3.2.2. Biodistribution of FAPI Tracer

The main excretory system for the FAPI PET tracer is the urinary system. Hence, there is robust accumulation of the FAPI tracer in the urinary bladder and urinary tract [79,80,81]. Interestingly, the excretion of some FAPI variants also includes the biliary system; therefore, uptake in the gallbladder and common bile duct was also observed [82]. Additionally, the uterus shows high uptake due to the high normal FAP expression in the uterus [79]. Moderate uptake of FAPI tracer was observed in the blood, pancreas, thyroid, liver, spleen, submandibular glands, Waldeyer’s ring, and myocardium. Minimal tracer accumulation was demonstrated in the brain, lung, oral mucosa, muscle, spine, and fat [80,82].

The time-dependent FAPI uptake pattern is distinct among different types of lesions. Malignant, inflammatory/reactive, and degenerative pathologies are distinguishable from one another by their TBR progression over time. With respect to [^68^Ga]Ga-FAPI-02, benign lesions present constant or slightly decreased uptake, whereas malignant lesions tend to present slight increase over time [82].

The uptake patterns of the different FAPI analogues are similar, but with subtle discrepancies. The accumulation of [^18^F]F-FAPI-42 was obvious in the gallbladder and common bile duct, impeding its use in detecting biliary diseases [80]. [^68^Ga]Ga-FAPI-46 demonstrated the most intensive accumulation in all pathologies, implying a better performance in malignant and non-malignant detection.

In a head-to-head comparative study of the biodistributions of [^68^Ga]Ga-FAPI and [^18^F]F-FDG, FAPI uptake was lower than FDG uptake in the brain, oral mucosa, parotid gland, myocardium, blood pool, liver, pancreas, spleen, kidney, spinal cord, and gastrointestinal tract. The uptakes of the two tracers were nearly equal in terms of the fat content. FAPI-avid showed a more significant increase than FDG-avid in the lungs, thyroid, and skeletal muscles. In summary, [^68^Ga]Ga-FAPI showed a better detection rate owing to the high TBR in lesions, especially those with high [^18^F]F-FDG background activity. Notably, [^68^Ga]Ga-FAPI accumulation in the mediastinal region, especially in the cardiac muscle and blood pool, was lower than that of [^18^F]F-FDG, implying that [^68^Ga]Ga-FAPI PET has a better diagnostic value than [^18^F]F-FDG in cardiovascular diseases [83].

## 4. Application of FAPI PET Imaging in Cardiovascular Diseases

### 4.1. Preliminary Evidence

Initially, FAPI uptake in cardiac diseases was discovered incidentally in cancer patients. Based on the largest cohort study of 229 patients with metastatic cancer, high [^68^Ga]Ga-FAPI uptake correlated with cardiovascular risk factors such as certain chemotherapies (platinum derivatives; OR = 3.0, *p* = 0.034), chest radiation history (OR = 3.5, *p* = 0.024), and metabolic disease [13]. 

The diagnostic potential of FAPI PET in cancer therapy-associated myocardial injury was reported in several studies [63,64,65]. Additionally, iatrogenic myocardial injury was also visualised using [^68^Ga]Ga-FAPI-46 PET in a retrospective study of 12 patients after pulmonary vein isolation [66]. Similarly, FAPI uptake in MI is incidentally observed in some patients with malignancies, indicating the potential feasibility of FAPI PET imaging for evaluating cardiac fibrosis after MI [45,46,49]. Furthermore, myocardial injury involved in systemic diseases such as cardiac amyloidosis and systemic sclerosis was revealed using [^68^Ga]Ga-FAPI-04 and [^68^Ga]Ga-FAPI PET imaging [57,67]. In addition, [^68^Ga]Ga-FAPI uptake in the right ventricle (RV) was observed in pressure overload cardiac diseases, such as idiopathic pulmonary arterial hypertension (IPAH) and hypertensive heart disease [54,56]. Likewise, investigation of FAPI PET in vascular diseases, such as thrombosis and atherosclerosis, was initiated because of accidental FAPI uptake in patients with various diseases [70,72,73,74].

The studies mentioned above provided preliminary evidence of the potential application of FAPI PET imaging in cardiovascular diseases, the details of which, especially in early detection, risk stratification, response evaluation, prediction of the evolution of LV function, and multimodal imaging, are discussed in the following sections.

### 4.2. Early Detection

Fibrosis is a common pathophysiological process that occurs during various circumstances that gradually becomes uncontrolled and excessive in cardiovascular diseases. Cardiovascular dysfunction is irreversible and fatal when fibrosis is established. Hence, early detection of fibrosis in cardiovascular diseases is of great significance.

#### 4.2.1. Myocardial Infarction

**Figure 2:**
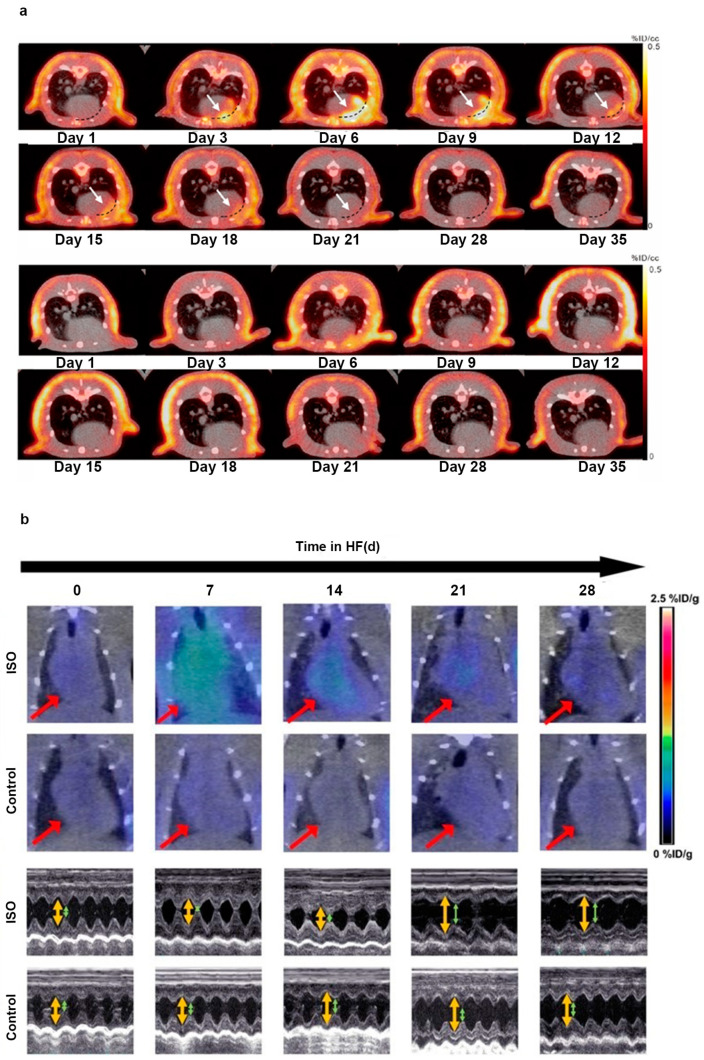
The application of FAPI PET in the early detection of cardiac disease. (**a**) [^68^Ga]Ga-FAPI-04 PET/CT images were obtained repeatedly in MI rats (the first and second rows) and sham-operated rats (the third and fourth rows) to evaluate the feasibility of FAPI PET in early detection of MI; (**b**) successive [^68^Ga]Ga-FAPI PET/CT images of the ISO-induced HF and control groups are displayed at 0, 7, 14, 21, and 28 d (the first and second rows). Corresponding transthoracic echocardiography is shown separately (the third and fourth rows). Adapted from Refs. [47,60].

Abrupt cessation of blood flow to the myocardium caused by coronary artery atherothrombosis can lead to MI, inducing cardiomyocyte necrosis and rearrangement of collagen fibres, serving as a potent trigger for myocardial replacement or reparative fibrosis [84]. Murine models of MI imaged with [^68^Ga]Ga-FAPI-04 PET showed that FAPI uptake in the infarct and adjacent regions increased, peaking at 6 days and gradually decreasing to the baseline level by 2 weeks, at different time points post-MI [44]. Moreover, FAPI uptake in the adjacent infarct region was more intense than in the infarct region. Radiolabelled FAPI represents a promising tracer for the in vivo imaging of fibroblast activation after MI. A recent animal MI model study emphasised the role of [^68^Ga]Ga-FAPI-04 PET/CT imaging in monitoring activated fibroblasts in the early post-MI stage and in evaluating reparative fibrosis [47] (Figure 2a). FAPI PET imaging can dynamically monitor reparative fibrosis after MI, which is beneficial for a better evaluation of the degree of reparative fibrosis in the early stages of MI and for guiding treatment. These studies underline the feasibility of FAPI imaging for tracing fibroblast activation and early visualisation of myocardial fibrosis post-MI. In concordance with these preclinical findings, a patient showed [^68^Ga]Ga-FAPI-04 uptake in the corresponding myocardium after acute MI, implying that the non-invasive assessment tool, FAPI PET imaging, may provide a great opportunity for the early identification of fibrosis [48].

#### 4.2.2. Heart Failure

HF is a clinical syndrome resulting from an abnormality in the structure or function of the heart, with 1-year and 5-year mortality rates of 20% and 53%, respectively, affecting at least 64.3 million people worldwide [85,86]. Disturbances in collagen metabolism, which is regulated by cardiac fibroblasts, play a vital role in the pathogenesis and progression of HF [87]. Fibroblast activation is a prerequisite for the repair and regeneration of progressive HF through remodelling of the ECM [17]. The early detection of myocardial remodelling and fibrosis is vital for monitoring and reversing the progression of HF.

The latest pre-clinical studies further explored the capacity of FAPI PET for the early detection of fibrosis in HF. Song et al. established an HF model using isoproterenol (ISO)-induced HF rats to collect sequential [^68^Ga]Ga-FAPI PET/CT images [60]. The phospho-autoradiography images demonstrated that the uptake of [^68^Ga]Ga-FAPI increased, peaked at the seventh day, and then decreased with time. In addition, the study recruited seven patients with HF and compared the clinical images of [^13^N]NH_3_ with those of [^68^Ga]Ga-FAPI PET/CT. As HF progressed, the FAPI uptake increased during the early stages (Figure 2b). This preclinical study demonstrated that FAPI PET imaging could be utilised to visualise activated fibroblasts, followed by myocardial remodelling and fibrosis, indicating that the early detection of FAP expression may be helpful for anti-fibrotic therapy. Recently, to determine the ability of [^68^Ga]Ga-FAPI-04 PET/CT to achieve early diagnosis of cardiac fibrosis in pressure overload HF, Wang et al. constructed a pressure overload HF model in rats that underwent abdominal aortic constriction (AAC) surgery [59]. The accumulation of [^68^Ga]Ga-FAPI-04 in the heart was higher in the AAC group than in the control group after the second week, whereas an echocardiographic abnormality appeared in the fourth week. In this comparative study, [^68^Ga]Ga-FAPI-04 PET has advantages in identifying the early stages of fibroblast activation and assessing the extent of cardiac fibrosis compared with echocardiography, which only reflects established cardiac fibrosis.

Pulmonary arterial hypertension (PAH) is a preliminary etiology of HF, which is characterised by overload pressure in the RV. Early detection with a novel strategy for optimal assessment of RV is warranted. A single-centre cohort enrolled 16 patients with PAH who underwent [^68^Ga]Ga-FAPI-04 PET/CT, echocardiography, and right heart catheterisation within 7 days to assess cardiac function and pulmonary haemodynamics, respectively [55]. The uptake of FAPI in the RV free wall (SUVmax: 2.5 ± 1.8, *p* < 0.001) and insertion site (SUVmax: 2.5 ± 1.7, *p* < 0.001) was significantly higher than in the LV (SUVmax: 1.5 ± 0.5) in 12 patients. In addition, the accumulation of FAPI in the RV free wall and insertion site was more significant in patients with tricuspid annular plane systolic excursion (TAPSE) < 17 mm than in those with TAPSE > 17 mm, demonstrating a relationship between FAPI uptake and RV dysfunction. The positive correlation between FAPI uptake and *n*-terminal pro-B-type natriuretic peptide (NT-proBNP) also supports the possibility of using FAPI PET/CT in the assessment of RV fibrotic remodelling. This pilot study demonstrated the potential of [^68^Ga]Ga-FAPI-04 PET/CT in the early detection of RV remodelling in patients with PAH, achieving better management of HF.

#### 4.2.3. Secondary Myocardial Injury

Myocardial fibrosis is a common response to various forms of secondary cardiac injuries, such as cancer treatment or cardiac surgery [20]. Cardiac fibroblasts play a central role in maintaining homeostasis of the ECM. During secondary myocardial injury, cardiac fibroblasts transition into the myofibroblast phenotype, resulting in cardiac fibrosis [87]. Cardiac fibrosis provokes a series of pathological changes ranging from cardiomyocyte hypertrophy and apoptosis to severe end-stage heart dysfunction. Timely identification of activated fibroblasts and ongoing fibrosis upon myocardial injury remains crucial for evaluating the progress and therapeutic window of the disease. FAPI PET is a novel imaging technique for early detection of fibrosis in secondary myocardial injury.

Totzeck et al. reported a case in which the accumulation of [^68^Ga]Ga-FAPI tracer was observed in the LV myocardium of a patient with pancreatic ductal cancer, with no clinical signs of cardiac discomfort, indicating secondary myocardial injury during chemotherapy [61]. This case demonstrates the utility of FAPI imaging for the early detection of fibrosis in secondary myocardial injury after chemotherapy and avoiding adverse cardiac events. A subsequent [^18^F]AlF-NOTA-FAPI-04 PET imaging study based on a retrospective analysis of patients with oesophageal squamous cell carcinoma and animal models confirmed that FAPI uptake in the damaged myocardium began to increase from the second week after radiation exposure, reaching a maximum on the fifth week [62]. Additionally, the LV ejection fraction (LVEF), measured using magnetic resonance imaging (MRI), decreased dramatically from week eight onwards. This study indicates that FAPI imaging has potential in a non-invasive incipient identification of fibrosis in radiation-induced myocardial injury compared to LVEF derived from MRI.

#### 4.2.4. Vascular Diseases

FAPI PET could achieve early detection of vascular diseases by visualising fibroblast activation and fibrosis [78]. A 28-year-old woman complained of shortness of breath for 2 months. Chest CT revealed an irregular soft-tissue mass in the mediastinum. [^18^F]F-FDG PET/CT showed diffuse FDG accumulation in the mediastinum and could not distinguish the lesion from the adjacent background. [^68^Ga]Ga-FAPI PET/CT indicated the lesion with intense FAPI uptake located in the main and bilateral proximal pulmonary arteries. Vascular involvement of Rosai–Dorfman disease was identified by histopathology of the resected lesion. Hence, [^68^Ga]Ga-FAPI PET/CT may outperform [^18^F]F-FDG PET/CT in the early detection of vascular lesions with a high TBR ratio through visualising fibroblast activation and fibrosis. 

A retrospective study with 69 oncologic patients was conducted to assess the feasibility of [^68^Ga]Ga-FAPI-04 PET/CT to detect arterial fibroblast activation [76]. FAPI uptake of the arterial wall in major vessel segments was investigated, the associations of which, with calcified plaque burden (including the number of plaques, plaque thickness, and calcification circumference), were then evaluated. A total of 64 out of 69 patients (92.8%) showed intense arterial [^68^Ga]Ga-FAPI-04 accumulation in 800 sites. Among these sites, 377 (47.1%) had vessel wall calcification that matched. Both the number of FAPI accumulation sites and the corresponding TBR had a significant correlation with the number, the thickness, and the circumference of calcified plaques. Therefore, [^68^Ga]Ga-FAPI-04 may be a potential tracer for early detection of arterial wall lesions and is correlated to marked calcification and overall calcified plaque burden.

### 4.3. Risk Stratification

Accurate risk stratification is crucial for formulating personalised and precise treatment regimens for cardiovascular diseases. FAPI PET imaging has advantages in the specific visualisation of activated fibroblasts as well as early and ongoing fibrosis, assisting in the appropriate evaluation of the activity and severity of cardiovascular disease.

#### 4.3.1. Coronary Artery Disease 

CAD, mostly attributed to atherosclerosis of the coronary artery, can lead to myocardial ischaemia and infarction. CAD is the leading cause of mortality worldwide and requires proper prevention and effective control strategies [88]. A critical pathophysiological basis of CAD is the combination of myocardial impairment and fibrosis replacement [87,89,90]. FAPI PET imaging may help dissect disease evolution and evaluate the activity and severity of fibrosis in CAD.

A retrospective thirty-two oncological patient cohort study indicated a significant correlation of CAD, age, and LVEF with remote standardised uptake value (SUV) mean uptake; of these, LVEF showed a very strong correlation with remote uptake (r^2^ = 0.74, *p* < 0.01), indicating the feasibility of [^68^Ga]Ga-FAPI-04 PET imaging in risk stratification in CAD [12]. 

A subsequent retrospective analysis investigated visual and quantitative myocardial [^18^F]AlF-NOTA-FAPI-04 uptake with various cardiovascular risk factors in a 21-oncological-patient cohort study [53]. A negative correlation was found between myocardial FAPI uptake and LVEF. The receiver operating characteristic (ROC) curve for predicting calcified plaques by myocardial FAPI uptake in the left anterior descending artery (LAD), left circumflex artery (LCX), and right coronary artery (RCA) territories showed areas under the curves (AUCs) of 0.786, 0.759, and 0.769, respectively. These findings are based on an unselected (potential subclinical CAD) population, which implies the utilisation of FAPI imaging for non-invasive CAD risk stratification and patient management. MI is a subtype of CAD with high mortality, and a rational disease assessment is urgently required. A retrospective cohort study of 10 patients who underwent [^68^Ga]Ga-FAPI-46 PET 18 ± 20.6 days after acute MI showed an inverse correlation between fibroblast activation volume and LV function (r = −0.69, *p* < 0.05) [46]. This study suggests that FAPI PET may provide meaningful insights into the risk stratification of cardiac fibrosis after MI.

#### 4.3.2. Cardiomyopathy

HCM is the most common genetically mutated cardiomyopathy and is attributed to sarcomeric protein gene mutations [91]. Patients with HCM have a higher risk of cardiac failure resulting from the activation of collagen-producing pathways and the early generation of a pro-fibrotic state [91,92]. The main pathophysiological changes in HCM are interstitial and perivascular fibrosis [93]. Myocardial fibrosis is associated with increased risk of HCM-related morbidity and mortality [94]. Thus, FAPI PET imaging may assist in risk stratification and patient management of HCM.

Only one study investigated FAPI PET in patients with HCM [69]. In this prospective study of 50 patients with HCM, myocardial [^18^F]AlF-NOTA-FAPI uptake was analysed according to intensity, extent, and amount. All HCM patients presented with increased and heterogeneous FAPI uptake in the LV myocardium compared with normal controls (median TBR, 8.8 vs. 2.1; *p* < 0.001). FAPI uptake regions were more than hypertrophic regions, as detected by CMR in HCM (median, 14 vs. 5, respectively; *p* < 0.001); 84% (interquartile range [IQR], 59–100%) of non-hypertrophic segments presented with FAPI uptake. Log-transformed FAPI amount had a negative correlation with LVEF z-score (−0.10; 95% CI: −0.21, −0.002). FAPI uptake was positively correlated with the 5-year sudden cardiac death (SCD) risk score (r = 0.32; *p* = 0.03). Therefore, FAPI PET may be a promising modality for risk stratification in HCM because it reflects myocardial fibrosis.

Light-chain cardiac amyloidosis (AL-CA) is a life-threatening disease involving multiple organs and is characterised by plasma cell dyscrasia with insoluble immunoglobulin AL fibril deposition in multiple organs and systems, leading to organ dysfunction [95]. The heart is the most involved organ and is associated with approximately one out of every two deaths. In cardiac amyloidosis (CA), amyloid deposition leads to cardiomyocyte necrosis and interstitial fibrosis [96]. Prompt detection and identification of CA-activated fibroblasts are important for both diagnosis and therapy, and may assist in the risk stratification of patients with CA. FAPI imaging may provide instructive evidence for therapy and patient stewardship [97].

**Figure 3:**
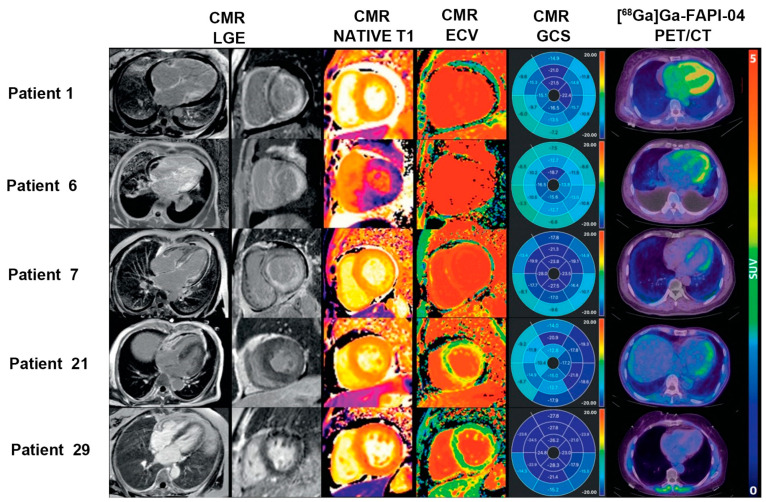
The application of FAPI PET in the risk stratification of cardiac disease. Three patients had CMR evidence of cardiac amyloid deposition with transmural late gadolinium enhancement (LGE), abnormally elevated T1 and ECV, and diffuse [^68^Ga]Ga-FAPI-04 uptake in the LV myocardium (Patient 1: extensive Mayo stage IIIb; Patient 6: extensive Mayo stage IIIa; and Patient 7: extensive Mayo stage II). Patient 21 (patchy Mayo stage II) showed non-diffuse LGE and focal uptake in the LV lateral wall on [^68^Ga]Ga-FAPI-04 PET imaging, while Patient 29 (negative Mayo stage I) had no LGE, but normal ECV and negative [^68^Ga]Ga-FAPI-04 uptake in the myocardium, indicating no cardiac involvement. Adapted from Ref. [68].

A recent prospective study of 27 patients with AL-CA showed that 80% (24/30) of patients presented with increased [^68^Ga]Ga-FAPI-04 LV uptake (patchy [*n* = 4] vs. extensive [*n* = 20]). SUV ratio and LV molecular volume were significantly higher in the PET-extensive group than in the PET-patchy group (2.79 mL ± 1.22 mL vs. 1.53 mL ± 0.66 mL [*p* = 0.045] and 198.3 mL ± 59.97 mL vs. 127.8 mL ± 25.82 [*p* = 0.005], respectively) [68] (Figure 3). Furthermore, FAPI uptake (LV SUVmean, SUVmax, and SUVratio) is significantly correlated with clinical biomarkers (Mayo stage and NT-proBNP), interventricular septal thickness, LVEF, LV end-systolic volume, extracellular volume (ECV), and LV global strain (all *p* < 0.05). Thus, this study demonstrated that FAPI imaging is a promising approach for assessing fibroblast activation in patients with AL-CA. Additionally, there was a significant correlation between FAPI uptake and disease severity, providing more information on the risk stratification of AL-CA.

#### 4.3.3. Atherosclerosis 

Atherosclerosis, an inflammatory disease of the arteries, is the predominant cause of cardiovascular diseases [98]. FAP is expressed by myofibroblasts derived from smooth muscle cells in human aortic plaques and is involved in the pathogenesis and progression of atherosclerosis, particularly in inflammatory processes [99,100]. The precise evaluation of atherosclerosis is important for risk stratification and patient management. A retrospective study quantified arterial fibroblast activation using [^68^Ga]Ga-FAPI-04 imaging in 1177 arterial segments from 41 patients without cardiovascular disease symptoms, correlating it with the degree of calcification and cardiovascular risk factors [75]. The FAPI TBR was negatively correlated with the degree of calcification, HU (r = −0.27, *p* < 0.01). Greater mean TBR was observed in higher-risk patients compared to that of lower-risk patients (2.2 ± 0.3 vs. 1.8 ± 0.3, *p* < 0.01). This study implies that FAPI PET imaging has potential as a feasible method for the non-invasive evaluation of fibroblastic activation in atherosclerosis, providing new perspectives into its pathogenesis and progression.

#### 4.3.4. Arteritis 

Takayasu arteritis (TA) is an idiopathic systemic inflammatory disease involving large arteries, including the aorta, its major branches, and pulmonary arteries [101]. Arterial inflammation is a key feature of TA and is characterised by thickening and remodelling of the arterial wall caused by myofibroblast proliferation [102]. Evaluating disease activity in patients with TA is challenging because there is no correlation between the clinical, biological, and radiological manifestations. A recent case report presented an example of [^68^Ga]Ga-FAPI PET/CT outperforming [^18^F]F-FDG PET/CT in evaluating disease activity and extent in a patient with TA [71]. Thus, FAPI PET imaging may have the potential to reliably assess disease activity and extent of TA, which is a prerequisite for the precise treatment of TA.

#### 4.3.5. Chronic Thromboembolic Pulmonary Hypertension

Chronic thromboembolic pulmonary hypertension (CTEPH) is a special type of pulmonary hypertensive disease, resulting from chronic pulmonary arterial occlusion by scar-like organised emboli that originated from an unresolved acute pulmonary embolism [103]. Fibrosis is essential for pathological pulmonary vascular remodelling [104]. A prospective study of [^68^Ga]Ga-FAPI-04 images of 13 patients with CTEPH and 13 controls analysed the correlation between FAPI uptake of the PA and remodelling parameters derived from right heart catheterisation (RHC) [77]. Visual interpretation revealed that nine of thirteen (69%) patients with CTEPH showed increased FAPI uptake, while none of the controls were positive. FAPI activity in the pulmonary vasculature (PA) was positively correlated with pulmonary arterial diastolic pressure (r = 0.571, *p* = 0.041). In conclusion, FAPI PET may be a feasible imaging modality for evaluating disease severity and improving the understanding of PA remodelling in CTEPH.

### 4.4. Response Evaluation

**Figure 4:**
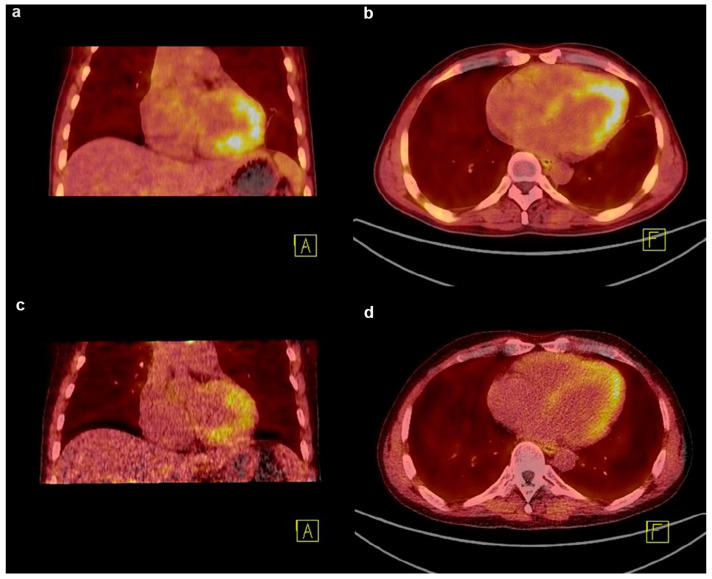
The application of [^18^F]F-FAPI PET in the response evaluation of cardiac disease. (**a**,**b**) FAPI PET/CT image of a patient with eosinophilic leukemia showing an intense uptake in the myocardium at baseline; (**c**,**d**) after 2 months of imatinib and prednisolone treatment, the follow-up FAPI PET image shows a decreased uptake in the corresponding area. Adapted from Ref. [58].

Eosinophilic leukaemia (EL) is a haematological disease characterised by persistent eosinophilia with variable manifestations [105]. Eosinophilic myocarditis (EM), a common complication in approximately 40–50% of patients with EL, is characterised by high morbidity and mortality [106]. EM usually involves necrotic, thrombotic, and fibrotic stages [107]. As fibrosis is a late stage of EM, effective intervention and accurate response evaluation during this period depend on the exact dissection of the fibrosis evolution. A case of a patient with EL and EM confirmed by bone marrow aspiration presenting with cardiac involvement, and evaluated by echocardiography and CMR, was reported [58]. Initially, imatinib and prednisolone were administered to treat EL. The first [^18^F]F-FAPI PET scan was performed to evaluate fibrosis before discharge, showing heterogeneous FAPI uptake in the myocardium and indicating underlying fibrosis with cardiac involvement. After 2 months of treatment, follow-up FAPI PET revealed that tracer uptake in the corresponding area was significantly reduced, implying mitigated and ameliorated fibrosis and favourable therapy response (Figure 4). Based on this study, FAPI-PET imaging can be used to formulate antifibrotic therapy and identify therapeutic responses in EM. It is speculated that FAPI PET imaging may be utilised in other fibrotic diseases other than in cardiovascular diseases to guide future anti-fibrotic treatment and response evaluation.

### 4.5. Prediction of the Evolution of LV Function 

**Figure 5:**
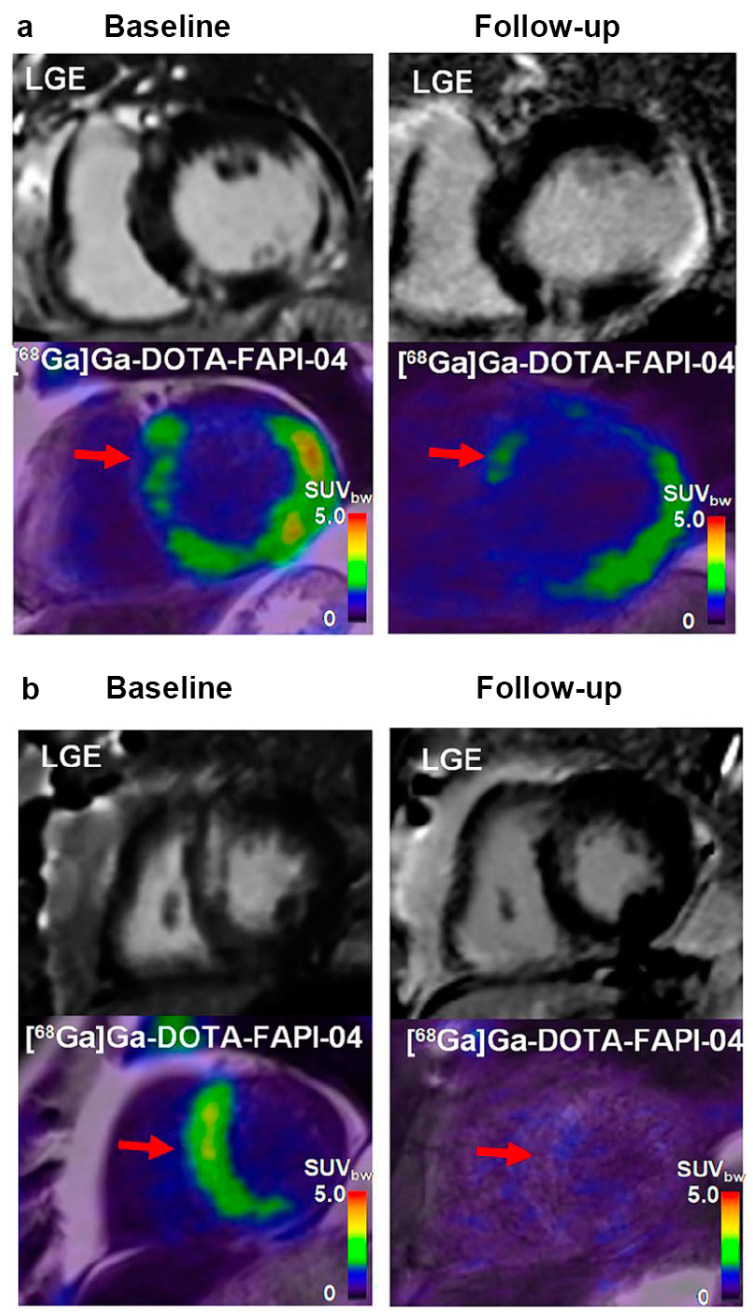
The application of [^68^Ga]Ga-DOTA-FAPI-04 PET in the prediction of the evolution of LV function. (**a**) In this patient (male, 66-year-old, with right coronary artery occlusion), large FAPI uptake volume (red arrow) was observed at both baseline and follow-up. Increased left ventricular end-diastolic volume (LVEDV) and left ventricular end-systolic volume (LVESV), and decreased LVEF indicated adverse LV remodelling. FAPI uptake is also shown in the remote non-LGE region (red arrow); (**b**) in another patient (male, 64-year-old, with left anterior descending coronary artery occlusion), small FAPI uptake volume (red arrow) at baseline and follow-up was observed. Decreased LVEDV and LVESV, as well as increased LVEF indicated better prognosis. Adapted from Ref. [52].

In cardiac diseases, myocardial fibrotic remodelling under various circumstances contributes to functional impairment, progressive dysfunction, and adverse events in the involved tissue. Accordingly, fibrosis is considered a credible therapeutic target that requires individualised strategies to tailor clinical decisions. Thus, FAPI PET imaging and non-invasive imaging with quantitative and fibrosis-targeted superiority may be useful for this purpose. A prospective [^18^F]AlF-NOTA-FAPI PET imaging study based on 14 first-time ST-segment elevation myocardial infarction (STEMI) patients after primary percutaneous coronary intervention (PPCI) identified the prognostic evaluation of FAPI PET imaging, post-MI, for the first time [51]. Baseline TBRmax was inversely correlated with LVEF at follow-up (r = −0.73, *p* = 0.02), implying that FAPI PET imaging may provide information regarding clinical outcomes in MI. A subsequent retrospective study investigated the correlation between CMR and [^68^Ga]Ga-FAPI-46 PET/CT in thirty-five patients with acute MI (AMI) [50]. The [^68^Ga]Ga-FAPI-46 signal did not match the manifestation of other imaging findings, but could predict the progression of cardiac dysfunction, complementary to current imaging techniques. Interestingly, the FAPI uptake volume was not correlated with LVEF measured simultaneously at baseline (r = −0.32, *p* = 0.07), whereas a significant inverse correlation between FAPI uptake volume and follow-up LVEF was observed (r = −0.58, *p* = 0.007). A recent prospective study quantitatively evaluated changes in the intensity and extent of myocardial fibroblast activation after AMI, using [^68^Ga]Ga-DOTA-FAPI-04 PET/MRI [52] (Figure 5a,b). The FAPI uptake volume was a better predictor (AUC = 0.938, *p* < 0.001) of late LV remodelling at 12 months after AMI than clinical characteristics, MR indexes, and cardiac functional parameters at baseline. In addition to its diagnostic ability, FAPI may also have a significant prognostic value.

## 5. Multimodal Imaging

### 5.1. FAPI vs. CMR

In cardiovascular diseases, a series of dynamic and complicated pathophysiological processes occurs, including inflammation, oedema, necrosis, and fibrosis. CMR is the preferred modality for non-invasive characterisation of myocardial lesions. CMR presents with myocardial injury by late gadolinium enhancement (LGE), fibrosis by native T1 relaxation times, and oedema by native T2 relaxation times [108]. In contrast to FAPI PET imaging, which reveals cellular signals originating from activated fibroblasts, CMR imaging primarily reveals extracellular tissue remodelling. However, the association between FAPI-PET and CMR is poorly understood. Briefly, the comparison revealed spatial and temporal mismatches between FAPI PET and CMR imaging. 

**Figure 6:**
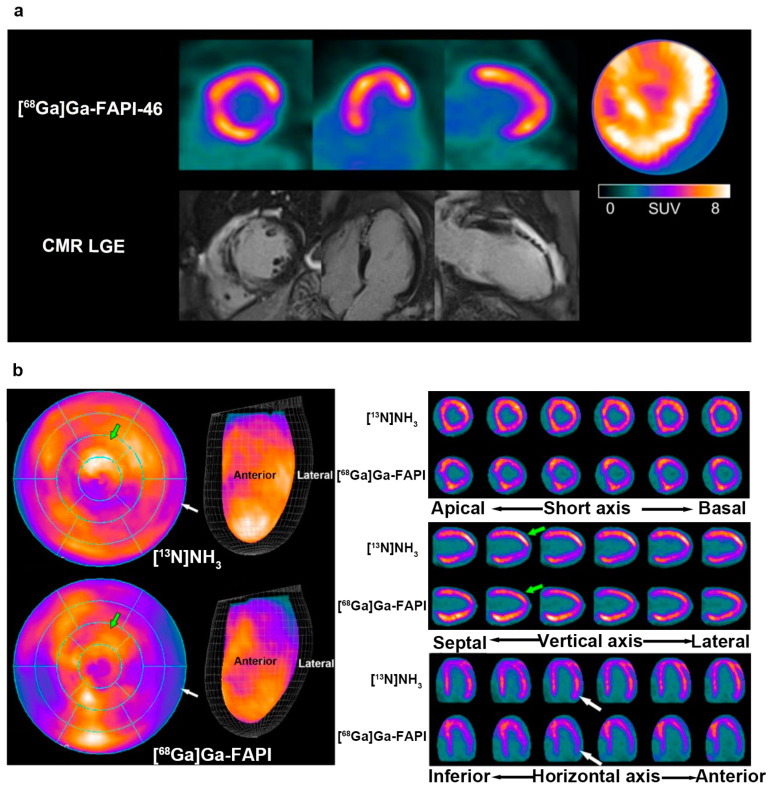
The comparative studies of FAPI PET and other modality imaging in cardiac disease. (**a**) The [^68^Ga]Ga-FAPI-46 PET uptake (the first row) region was larger than for the LGE signal from CMR (the second row) in a patient with acute myocardial infarction; (**b**) a 31-year-old male with liver sarcoma underwent [^13^N]NH_3_ and [^68^Ga]Ga-FAPI after immunotherapy. The uptake of [^13^N]NH_3_ was decreased in the LV, while that of [^68^Ga]Ga-FAPI accumulated instead. Adapted from Refs. [50,65].

To date, only a few studies examined the underlying relationship between these factors in limited cardiovascular diseases. A prospective study that recruited 14 STEMI patients revealed that [^18^F]AlF-NOTA-FAPI uptake was associated with myocardial injury biomarkers’ T2-weighted imaging (T2WI), ECV, and LGE derived from CMR at both patient and segmental levels (all *p* < 0.05) [51]. Additionally, the areas of focal and heterogeneous FAPI accumulation were larger than those observed in the CMR images. This suggests that FAPI PET imaging surpassed CMR imaging in detecting the myocardium involved in reperfused STEMI. Similarly, a retrospective study of 35 patients with AMI proved that the area of [^68^Ga]Ga-FAPI-46 uptake markedly exceeded the infarcted area that was detected on CMR, suggesting that the cell-based FAPI uptake of fibroblast activation differed from CMR-derived interstitial features [50]. Moreover, a significant inverse correlation was observed between FAPI-PET and follow-up LVEF. It is speculated that the mismatch between FAPI uptake and CMR imaging after AMI demonstrates the potential of FAPI imaging to complement CMR imaging in predicting the progression of ventricular dysfunction and remodelling (Figure 6a). In addition to MI, FAPI PET imaging and CMR were also compared in cardiomyopathy. In a prospective study of 50 patients with HCM, [^18^F]AlF-NOTA-FAPI accumulation regions were more than the hypertrophic regions detected by CMR (median, 14 vs. 5, respectively; *p* < 0.001); and 84% (IQR, 59–100%) in non-hypertrophic segments that presented with FAPI uptake [69]. Hence, the myocardial area involved in FAPI PET imaging was larger than the hypertrophic area in CMR imaging, and this was attributed to the distinct pathophysiology of fibroblast activation from hypertrophy in HCM. 

Additionally, the temporal mismatch between FAPI-PET and CMR is noteworthy. A recent animal study and retrospective analysis of patients with oesophageal squamous cell carcinoma confirmed that [^18^F]AlF-NOTA-FAPI-04 uptake in the damaged myocardium began to increase from the second week after radiation exposure and reached a maximum at the fifth week, whereas LVEF measured by CMR decreased from week eight onwards [62]. The earlier manifestation of abnormalities in FAPI PET imaging indicates its potential to assist CMR in the detection of radiation-induced myocardial injury. 

### 5.2. FAPI vs. [^68^Ga]Ga-DOTATATE

Somatostatin receptors (SSTR) are proteins that are overexpressed in neuroendocrine tumours [109]. Five subtypes of SSTR (SSTR1–5) were identified to date, among which, SSTR2 is highly expressed in neuroendocrine tumours and is an ideal target molecule for radiometric imaging [110]. SSTR2 was also identified as an indicator of activated macrophages in lesions that are associated with inflammation [111,112]. Hence, in addition to its application in malignancies, [^68^Ga]Ga-DOTATATE recently attracted significant interest for the visualisation of inflammation in cardiovascular diseases [113], MI [114], and more recently, in acute myocarditis [115].

A patient with advanced upper tract urothelial carcinoma treated with ICIs was enrolled in a dual-tracer imaging modality study because of suspected cardiac dysfunction [64]. The images of [^68^Ga]Ga-DOTATATE and [^68^Ga]Ga-FAPI-04 were obtained on two separate days. [^68^Ga]Ga-DOTATATE PET manifested scarce, diffused, and homogenous tracer accumulation at 87% of the LV (TBR: 2.65, range: 1.85–3.47). On the contrary, FAPI PET showed intense, focal, and heterogeneous accumulation in the septal, anterior, and lateral wall rather than in the inferior wall at 52% of the LV (TBR: 1.36, range: 1.09–1.77). The mismatched images between [^68^Ga]Ga-DOTATATE and [^68^Ga]Ga-FAPI-04 indicate the potential of FAPI PET to distinguish certain pathophysiological processes other than inflammation in ICI-associated myocarditis.

### 5.3. FAPI vs. [^13^N]NH_3_

^13^N-ammonia ([^13^N]NH_3_) is a classic tracer used in cardiac PET imaging to evaluate blood flow in the myocardium and coronary arteries [116,117,118]. Interestingly, a patient with liver sarcoma after immunotherapy underwent two PET investigations using [^13^N]NH_3_ PET and [^68^Ga]Ga-FAPI PET [65]. The former manifested a heterogeneously decreased uptake in the LV, whereas an increased and uneven accumulation of [^68^Ga]Ga-FAPI was observed in the anterior and inferior walls (Figure 6b). The mismatch between the images of the two different tracers suggested an inconsistency in myocardial perfusion and injury caused by ICI-associated myocarditis, unlike that of MI, implying the potential of FAPI imaging in the differential diagnosis of myocardial injury.

## 6. Future Directions

Fibrosis is a common response to stress and injury in cardiovascular diseases. The evolution of fibrosis contributes to adverse cardiovascular remodelling and adverse cardiovascular events. Fibroblast activation is the key point for the formation and progression of fibrosis. The incipient detection and evaluation of activated fibroblasts is essential for patient management and prognosis. FAP, as a biomarker of activated fibroblasts, was targeted and visualised by FAPI PET to reflect and evaluate fibrosis in cardiovascular diseases. 

The state-of-the-art applications and the performance of FAPI PET in various cardiovascular diseases were summarised and discussed. Initially, FAPI PET in cancer therapy-associated myocardial injury, iatrogenic myocardial injury, myocardial injury involved in systemic diseases such as cardiac amyloidosis and systemic sclerosis, and vascular diseases such as thrombosis and atherosclerosis were incidentally discovered and preliminary investigated. Significantly, the potential of FAPI PET in tracing fibroblast activation and accomplishing early detection of cardiovascular fibrosis was evaluated and identified in MI, myocardial remodelling in HF, secondary myocardial injury, and vascular diseases. Furthermore, FAPI PET could assist in risk stratification on CAD, cardiomyopathy, atherosclerosis, arteritis, and CTEPH through appropriate evaluation of the activity and severity of cardiovascular disease. Furthermore, the role of FAPI PET in response evaluation of cardiac disease was also demonstrated in a patient with EL and EM. In addition, the prognostic value of FAPI PET was also investigated in MI, and supposed as a recommendable modality for prediction of the evolution of LV function.

Comparative and complementary studies on multimodal imaging were conducted. The mismatch between FAPI uptake and CMR imaging demonstrates the potential of FAPI imaging to complement CMR imaging in predicting remodelling in MI, depicting the extent of fibrosis apart from hypertrophy in HCM, and earlier detection of radiation-induced myocardial injury. Moreover, FAPI PET combined with other imaging modalities is beneficial for distinguishing certain pathophysiological processes and differential diagnosis.

Although FAPI PET is considered as a promising tool for evaluating cardiovascular fibrosis, there are still gaps in knowledge and studies needing filling. Firstly, the potential of FAPI PET for early detection of vascular disease is little tapped. Secondly, given the limited number and volume of studies on FAPI PET imaging in early detection and risk stratification for cardiovascular diseases, large-population and multicentre studies are warranted to maximise the optimal capacity of FAPI PET imaging in these fields. Thirdly, future research perspectives on FAPI PET imaging in cardiovascular diseases could lay particular emphasis on response evaluation and prediction of the evolution of LV function, as there are merely a few related studies with small-volume and single-sort disease to date. Thirdly, the comparison and relationship between FAPI PET imaging and other imaging modalities should be further investigated to better understand the advantages and optimised application scenarios of FAPI PET imaging. Lastly, due to the current application of FAP inhibitors in cancer treatments [119], FAPI-PET imaging opens the door to a new theranostic approach to cardiovascular diseases. Hence, limiting the fibroblast activity in various cardiovascular diseases should also be investigated in the future.

## 7. Conclusions

Fibrosis is a response to various cardiovascular pathologies that manifests as ECM remodelling and superfluous collagen deposition mediated by activated fibroblasts. FAP is specific for identifying activated fibroblasts, which are biomarkers of the early stages of cardiovascular fibrosis. The FAP-targeting radiotracer FAPI PET imaging may be used for specific imaging of incipient fibrosis in cardiovascular diseases. This approach may facilitate the identification of early fibrosis, distinguish early-stage vs. end-stage diseases, evaluate therapeutic responses, and predict cardiac function in cardiovascular diseases. 

## Figures and Tables

**Table 1:** Summary of FAPI PET studies in cardiovascular diseases.

Diseases	Year	Radiopharmaceutical	Participants	Study Design	Authors	Summary	Ref.
Myocardial infarction	2019	[^68^Ga]Ga-FAPI-04	-	Pre-clinical	Varasteh et al.	[^68^Ga]Ga-FAPI-04 imaging of activated fibroblasts after MI have diagnostic and prognostic value for clinical management of patients after MI.	[44]
	2020	[^68^Ga]Ga-FAPI-04	1	Case	Zhu et al.	[^68^Ga]Ga-FAPI-04 accumulation in the inferior wall of LV was incidentally found and diagnosed as myocardial infarction in a patient with neuroendocrine carcinoma.	[45]
	2021	[^68^Ga]Ga-FAPI-46	10	Retrospective	Kessler et al.	The [^68^Ga]Ga-FAPI-46 uptake area coincided well with identified culprit lesion by coronary angiography. Fibroblast activation volume was strong correlated with LV function and peak creatine kinase.	[46]
	2022	[^68^Ga]Ga-FAPI-04	-	Pre-clinical	Qiao et al.	The activated fibroblasts could be traced by [^68^Ga]Ga-FAPI-04 PET in the early stage after acute MI, evaluating the degree of reparative fibrosis rather than reactive fibrosis.	[47]
	2022	[^68^Ga]Ga-FAPI-04	5	Case	Notohamiprodjo et al.	The uptake of [^68^Ga]Ga-FAPI-04 extends beyond the actual infarcted area and overestimates the infarct size as confirmed by follow-up CMR.	[48]
	2022	[^68^Ga]Ga-FAPI	1	Case	Yuan et al.	The [^68^Ga]Ga-FAPI uptake was in good concordance with abnormal CMR signals and high FDG uptake in a post-MI patient, showing its potential to evaluate activated fibroblast and cardiac remodelling after MI.	[49]
	2022	[^68^Ga]Ga-FAPI-46	35	Retrospective	Diekmann et al.	The uptake of [^68^Ga]Ga-FAPI-46 mismatched the characteristics of myocardial tissue unveiled by CMR; the FAPI PET signal was feasible to predict the progression of ventricular dysfunction.	[50]
	2022	[^18^F]AlF-NOTA-FAPI	28	Prospective	Xie et al.	The [^18^F]AlF-NOTA-FAPI PET showed advantages over CMR in detecting involved myocardium in reperfused STEMI, and was correlated with follow-up LVEF and myocardial damage.	[51]
	2023	[^68^Ga]Ga-DOTA-FAPI-04	26	Prospective	Zhang et al.	The [^68^Ga]Ga-DOTA-FAPI-04 uptake volume in the LV remodelling group after STEMI was bigger than that of the non-LV remodelling group at baseline, which significantly predicted late LV remodelling.	[52]
CAD	2022	[^18^F]AlF-NOTA-FAPI-04	21	Retrospective	Lyu et al.	The [^18^F]AlF-NOTA-FAPI-04 uptake was correlated with cardiovascular risk factors and the distribution of coronary plaques.	[53]
CAD and heart failure	2021	[^68^Ga]Ga-FAPI-04	32	Retrospective	Siebermair et al.	There was significant correlation of CAD, age, and left ventricular ejection fraction (LVEF) with remote [^68^Ga]Ga-FAPI-04 uptake, especially LVEF.	[12]
Heart failure	2022	[^68^Ga]Ga-FAPI	1	Case	Wang et al.	The [^68^Ga]Ga-FAPI uptake was observed in the right heart in a patient with pulmonary arterial hypertension for the first time.	[54]
	2022	[^68^Ga]Ga-FAPI-04	16	Prospective	Gu et al.	There was significant positive correlation between cardiac [^68^Ga]Ga-FAPI-04 uptake and parameters of pulmonary hemodynamics and cardiac function in pulmonary arterial hypertension patients.	[55]
	2022	[^68^Ga]Ga-FAPI	1	Case	Lin et al.	This case indicated hypertensive heart disease, which is characterised by myocyte disarray and fibrosis, had active [^68^Ga]Ga-FAPI uptake.	[56]
	2022	[^68^Ga]Ga-FAPI-04	14	Prospective	Treutlein et al.	Dynamic changes of [^68^Ga]Ga-FAPI-04 uptake were associated with changes in the activity of systemic sclerosis-related myocardial fibrosis.	[57]
	2022	[^18^F]F-FAPI	1	Case	Si et al.	[^18^F]F-FAPI PET achieved the visualisation of activated fibroblast at early stage of EM, and may aid tailored antifibrotic therapy toward EM patients.	[58]
	2023	[^68^Ga]Ga-FAPI-04	-	Pre-clinical	Wang et al.	The activated fibroblasts in the heart and liver after pressure overload can be monitored by [^68^Ga]Ga-FAPI-04 PET/CT, which reveals an early fibrotic link in cardio–liver interactions and could better predict nonischemic heart failure prognosis.	[59]
	2023	[^68^Ga]Ga-FAPI	-	Pre-clinical	Song et al.	As heart failure progresses, [^68^Ga]Ga-FAPI uptake is high in the early stage and then gradually decreases. Active myocardial FAP expression is followed by myocardial remodelling and fibrosis.	[60]
Chemotherapy-induced myocardial injury	2020	[^68^Ga]Ga-FAPI	1	case	Totzeck et al.	[^68^Ga]Ga-FAPI PET/CT may be a promising modality to early detect myocardial damage after chemotherapy for cardiotoxicity prevention.	[61]
	2020	[^68^Ga]Ga-FAPI	229	Retrospective	Heckmann et al.	High [^68^Ga]Ga-FAPI signal intensities correlate with cardiovascular risk factors and metabolic disease.	[13]
Radiation-induced myocardial injury	2023	[^18^F]AlF-NOTA-FAPI-04	-/13	Pre-clinical and retrospective	Wei et al.	[^18^F]AlF-NOTA-FAPI-04 PET tracer can be applied in the detection of radiation-induced myocardial damage, which obtained earlier exhibition than the decrease in LVEF.	[62]
Immune checkpoint inhibitors (ICIs)-associated myocardial injury	2021	[^68^Ga]Ga-FAPI	26	Retrospective	Finke et al.	[^68^Ga]Ga-FAPI PET/CT may be used to identify ICI-associated myocarditis patients at an early stage. Moreover, FAPI PET/CT also contributes to cardiac risk stratification besides biomarker, ECG, and echocardiography when integrated into cancer stage diagnostics.	[63]
	2022	[^68^Ga]Ga-FAPI-04	1	Case	Niu et al.	The mismatched image between [^68^Ga]Ga-DOTATATE PET and [^68^Ga]Ga-FAPI-04 PET revealed multiple cardiac molecular injuries induced by ICIs.	[64]
	2023	[^68^Ga]Ga-FAPI	1	Case	Zhang et al.	The areas of myocardial injury and fibrosis caused by ICI-associated myocarditis may be inconsistent. [^68^Ga]Ga-FAPI PET imaging may be used to detect ICI-associated myocarditis-induced heart failure, serving as indirect evidence of fibroblast activation.	[65]
Iatrogenic myocardial injury	2022	[^68^Ga]Ga-FAPI-46	12	Retrospective	Kupusovic et al.	[^68^Ga]Ga-FAPI-46 PET/CT visualised fibroblast activation of thermal damage after pulmonary vein isolation. Cryoballoon ablation seems to cause more pronounced fibroblast activation following tissue injury than radiofrequency.	[66]
Cardiac amyloidosis	2022	[^68^Ga]Ga-FAPI	1	Case	Guo et al.	[^68^Ga]Ga-FAPI PET showed diffuse and inhomogeneous accumulation in the thickened LV myocardium in a patient suspected with cardiac amyloidosis, indicating its potential in early detection of cardiac amyloidosis.	[67]
	2022	[^68^Ga]Ga-FAPI-04	30	Prospective	Wang et al.	[^68^Ga]Ga-FAPI-04 PET/CT is feasible in detecting myocardial fibroblast activation in patients with systemic amyloid light chain cardiac amyloidosis in correlation with myocardial remodelling. It might provide complementary information on cardiac molecular characterisation and staging of disease.	[68]
HCM	2023	[^18^F]AlF-NOTA-FAPI	72	Prospective	Wang et al.	[^18^F]AlF-NOTA-FAPI PET/CT imaging indicated intense and heterogeneous activity in hypertrophic cardiomyopathy, and FAPI uptake was associated with 5-year risk of sudden cardiac death.	[69]
Thrombosis	2021	[^68^Ga]Ga-FAPI	1	Case	Lin et al.	[^68^Ga]Ga-FAPI PET/CT showed multiple increased accumulation in the cerebral venous sinus and diagnosed as neuro-Behçet disease finally.	[70]
Arteritis	2021	[^68^Ga]Ga-FAPI	1	Case	Wu et al.	This case presented an example where [^68^Ga]Ga-FAPI outperformed [^18^F]F-FDG for detecting active inflammation of Takayasu arteritis. Therefore, [^68^Ga]Ga-FAPI PET/CT may allow monitoring of disease activity.	[71]
Atherosclerosis	2022	[^68^Ga]Ga-FAPI	1	Case	Hu et al.	The case presented an increased [^68^Ga]Ga-FAPI uptake was observed in active coronary atherosclerotic plaque and the esophageal cancer with esophagitis.	[72]
	2022	[^68^Ga]Ga-FAPI	1	Case	Yang et al.	This case showed [^68^Ga]Ga-FAPI-avid plaque of basilar artery accounting for the embolic events resulting in downstream infarction. Thus, FAPI imaging may have potential for detection of vulnerable plaques.	[73]
	2022	[^68^Ga]Ga-FAPI-04	137	Retrospective	Qi et al.	The retrospective study included 392 non-tumoural uptake regions in [^68^Ga]Ga-FAPI-04 PET images. Ten regions with high FAPI uptake were confirmed as atherosclerosis.	[74]
	2022	[^68^Ga]Ga-FAPI-04	41	Retrospective	Wu et al.	Increased expression of FAP in fibrous caps may contribute to progression of atherosclerotic plaques. [^68^Ga]Ga-FAPI-04 PET/CT may be potential for imaging fibroblastic activation in the arterial wall.	[75]
	2023	[^68^Ga]Ga-FAPI-04	69	Retrospective	Kosmala et al.	[^68^Ga]Ga-FAPI-04 PET revealed arterial wall lesions and correlated with marked calcification and overall calcified plaque burden, but is not consistently associated with cardiovascular risk.	[76]
CTEPH	2022	[^68^Ga]Ga-FAPI-04	26	Prospective	Gong et al.	[^68^Ga]Ga-FAPI-04 demonstrated the potential for visualising fibroblast activation in the pulmonary artery wall, and the tracer uptake was positively correlated with pulmonary arterial diastolic pressure.	[77]
Secondary vascular injury	2022	[^68^Ga]Ga-FAPI	1	Case	Zhao et al.	[^68^Ga]Ga-FAPI PET/CT may outperform [^18^F]F-FDG PET/CT in the early detection of vascular lesions with a high target-to-background ratio.	[78]

## Data Availability

Not applicable.
